# Supplementary material for: A large meta-analysis identifies genes associated with anterior uveitis
Source: Nat Commun. 2023 Nov 11;14:7300. doi: 10.1038/s41467-023-43036-1 (PMC10638276; doi:10.1038/s41467-023-43036-1)
Supplement: Supplementary file 1 — Supplementary Information [file 41467_2023_43036_MOESM1_ESM.pdf]

A Large Meta-Analysis Identifies Genes Associated with Anterior Uveitis

Gelfman et al. 2023

Supplementary Information

Results

Rare variant analyses identify novel genes contributing to AU risk

IPMK variants

The top association consisted of 11 AU carriers and 411 controls. The aggregated case variants included ten distinct pLoF and missense variants (6 missense, 2 stop-gain, 2 frame-shifts), none of which were significant independently, but all of which are expected to damage the protein function (supplementary Data 1-2).

IDO2 variants

The top association consisted of 28 AU cases (27 Het and 1 Hom) and 1,438 controls (1433/5). The aggregated variants that constructed the association included seven distinct pLoF variants, four stop-gain, two frame-shifts and one splice-donor (supplementary Data 2-3).

Additional testing of candidate genes

Due to the small number of carriers, we complemented the rare gene-burden analysis with a Fishers Exact Test (FET). While not adjusting for the complex population structure of the cohorts, the FET test added another level of information for consideration of the findings that have low number of carriers. In addition, the test was performed on the aggregated number of variants from all-cohorts, similar to a mega-analysis, disregarding replication of signals between the cohorts. The FET results for the three borderline significant genes showed weaker OR and p-value: *ACHE* showed a reduced, yet still high OR=8.89, and weaker p=8.27e-05. In addition, all six *ACHE* carriers were of EUR ancestry (supplementary figure S12A). For *STXBP2*, we found a reduced confidence FET (OR=7.67, p=4.66e-06). Surprisingly, all nine cases were of AFR descent, which might point to an ancestry specific effect (supplementary figure S12B). However, six out of the nine carriers originate from the small UPENN cohort and do not replicate within the other cohorts. Last, *ADGRF5* showed a similar reduction in FET OR and p-value (OR=8.28, p=4.44e-04), an expected result considering the low number of carriers that

were of both AUR and AFR ancestries (Figure S12C). A detailed annotated information of all variants that are incorporated into the top gene burdens is presented in supplementary Data 2. In summary of the above, FET analysis suggested that additional support is required for the genes with low case count for nomination as novel risk genes for AU.

Gene burden analyses of B*27-neg AU

We found that both *IPMK* and *IDO2* replicated a similar direction of risk in the B*27-neg cohort: the same *IPMK* model that included pLoF and missense variants that are strongly deleterious (predicted by 5/5 prediction models), with AF <0.1%, showed a similar significance with OR[95% CI]=10.31 [4.8-22.2] and p=2.36e-09. This is due to the previous eleven case carriers all belonging to the B*27-neg group, while control samples were decreased to 381 (Table 4). For IDO2, the rare pLoF mask (AF<0.1%) showed a similar effect with OR[95% CI]=3.28 [2.04 – 5.27], but a weaker p=9.5e-07 owing to the decreased number of samples. Interestingly, 24 of 28 IDO2 pLoF case carriers are of African (AFR) ancestry, a significantly larger proportion of AFR carriers than their proportion in the case cohort (~5%). These include 19 carriers of stop-gain rs151088117 (8:39989793:A:T) and 4 carriers of stop-gain rs199869245 (8:39989787:C:T). The allele frequency of rs151088117 in AFR population is AF=0.0107 calculated based on 22,440 AFR alleles in the gnomAD database; the same SNP is very rare in non-finish EUR (AF=0.0) considering 121,344 alleles [1]. Since rs151088117 is more borderline common in AFR population high, a similar rare variant analysis excluding variants with AF<0.1% in AFR population will not yield similar results. That said, AFR only analyses considering all ancestry AF filter yielded a similar risk as in EUR (OR~=2-3).

References

1 Karczewski, K. J. *et al.* The mutational constraint spectrum quantified from variation in 141,456 humans. *Nature* **581**, 434-443, doi:10.1038/s41586-020-2308-7 (2020).

Supplementary Figures

Supplementary Figure S1. Common *HLA-B* risk and *ERAP1* protection with 3,180 European AU cases and 826,685 European controls. A Manhattan plot depicting the -log10(P-value) for all common variants (y-axis) across all chromosomes (x-axis). *HLA-B* top risk signal is shown by an upward triangle on chromosome six, while *ERAP1* protection is shown by the downward triangle on chromosome five. Association models were run with age, age^2, sex and age × sex, and 10 ancestry-informative principal components as covariates. P-values are uncorrected and are from two-sided tests performed using approximate Firth logistic regression.

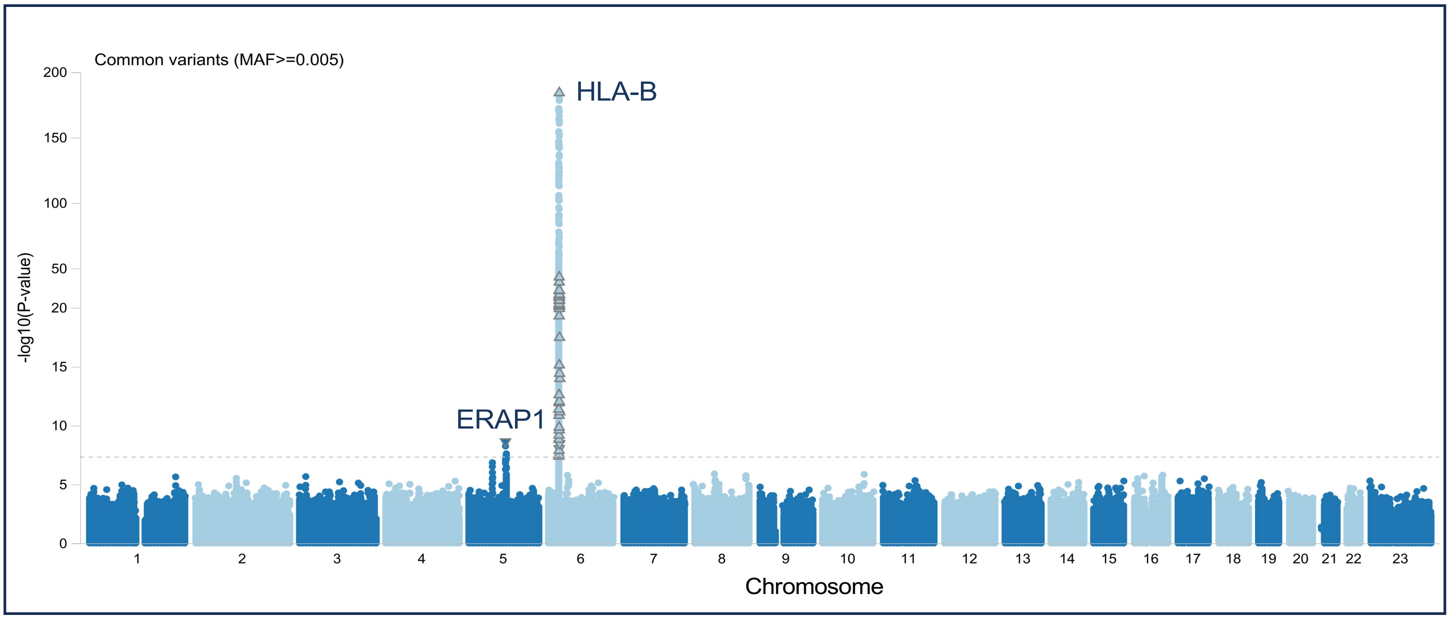

Supplementary Figure S2. Top SNPs at the *HLA-B* and *ERAP1* loci across eight cohorts when considering only European samples. A) A forest plot depicting the association details for *HLA-B* top risk variant rs543685299 in each of the eight cohorts tested including only EUR ancestry. **B)** A forest plot depicting the association details for the top *ERAP1* protective intronic variant rs3198304 in the eight cohorts tested and including only EUR ancestry. A meta-analysis result combining all cohorts is the lowest row (bold), meta-analysis OR is presented by a red diamond. Centre points represent odds ratios as estimated by approximate Firth logistic regression, with errors bars representing 95% confidence intervals. P-values are uncorrected and reflect two-sided tests. Numbers below the cases and controls columns represent counts of individuals with homozygote reference, heterozygote and homozygous alternative genotypes, respectively.

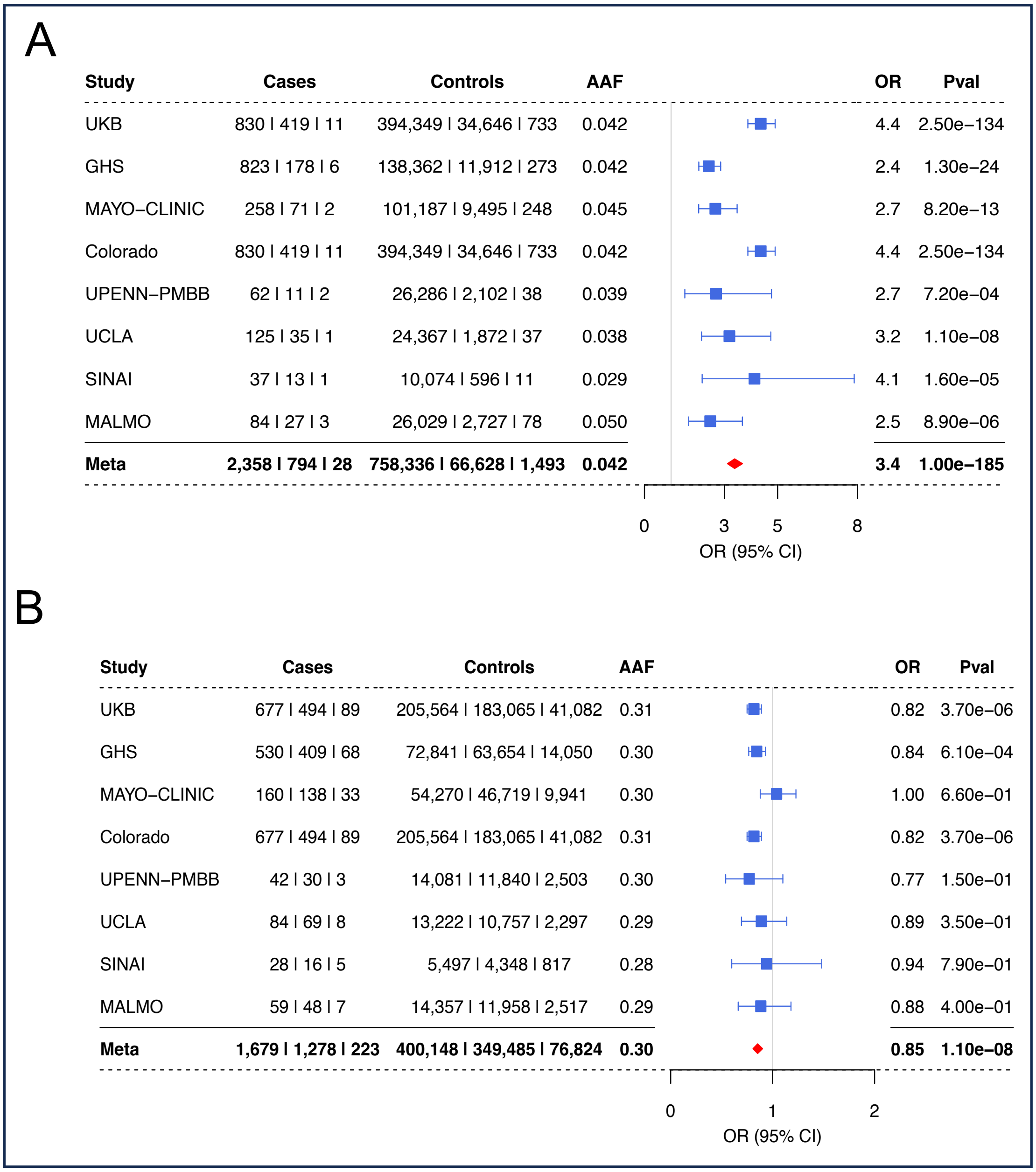

Supplementary Figure S3. Gene burden results combining all eight cohorts including with 3,850 AU cases and 916,549 controls. The gene burden analyses combining all cohorts exhibit a controlled low inflation of ʎ=0.935 (top right panel). Top gene burdens for *IPMK* and *IDO2* are labeled with red tringle indicating p-value and direction of effect. Dashed line represents a p-value of 5.0e-08. Association models were run with age, age^2, sex and age × sex, and 10 ancestry-informative principal components as covariates. P-values are uncorrected and are from two-sided tests performed using approximate Firth logistic regression.

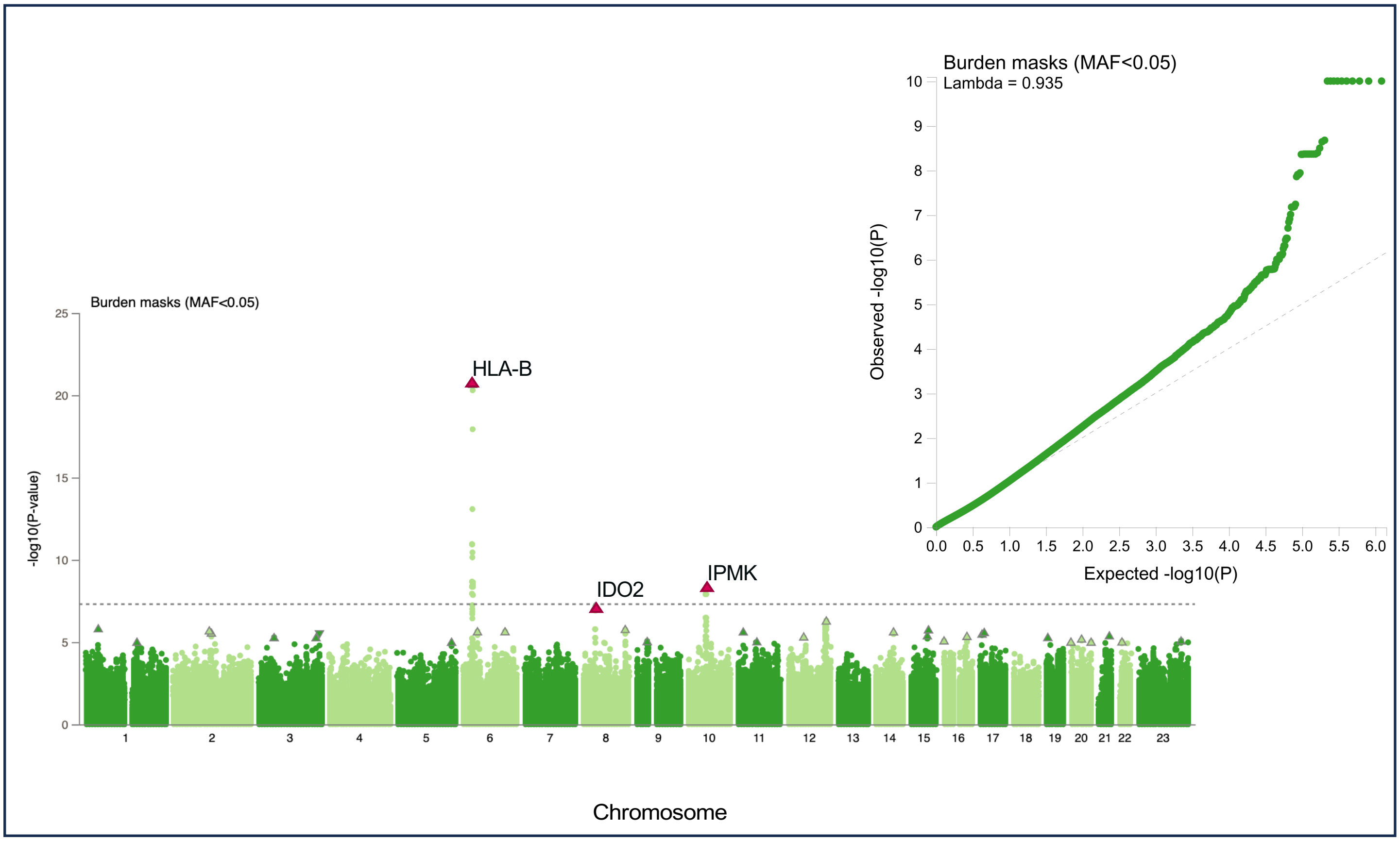

Supplementary Figure S4. Conditional analysis using *HLA-B* tagging SNP rs4349859. A Manhattan plot depicting the -log10(P-value) for all common variants (y-axis) across all chromosomes (x-axis). *ERAP1* significant protective signal is shown by a downward triangle on chromosome five. A borderline *HLA-DPB1* risk signal is also labeled. Dashed line represents a p-value of 5.0e-08. Association models were run with age, age^2, sex and age × sex, and 10 ancestry-informative principal components as covariates. P-values are uncorrected and are from two-sided tests performed using approximate Firth logistic regression.

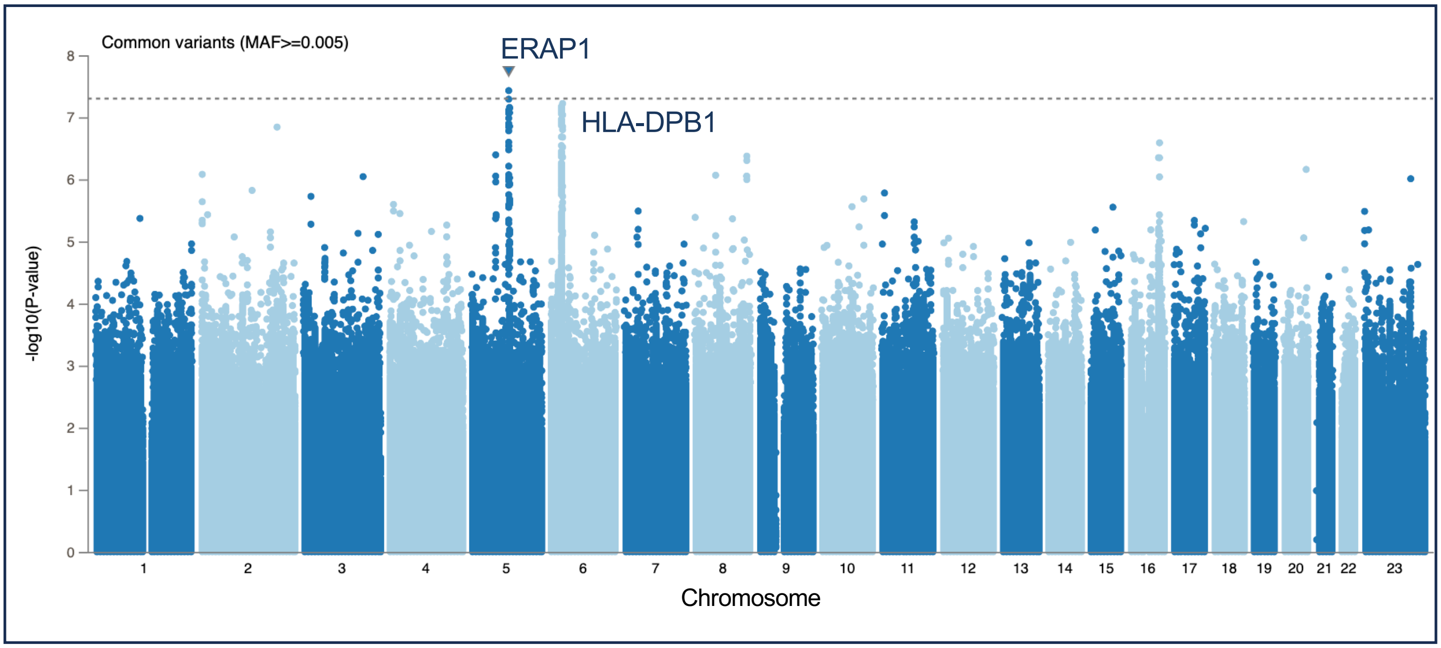

Supplementary Figure S5. Top *ERAP1* SNP in a B*27-stratified analysis. A forest plot depicting the association details for *ERAP1* top risk variant (rs30187) in all cohorts tested when considering only EUR samples. A meta-analysis result combining all cohorts is the lowest row (bold), meta-analysis OR is represented by a red diamond. The EUR-only, B*27-pos analysis confirmed the significant results for the *ERAP1* locus with OR [95% CI] = 0.73 [0.66–0.81], and a p=4.1e-10 for rs30187. Centre points represent odds ratios as estimated by approximate Firth logistic regression, with errors bars representing 95% confidence intervals. P-values are uncorrected and reflect two-sided tests. Numbers below the cases and controls columns represent counts of individuals with homozygote reference, heterozygote and homozygous alternative genotypes, respectively.

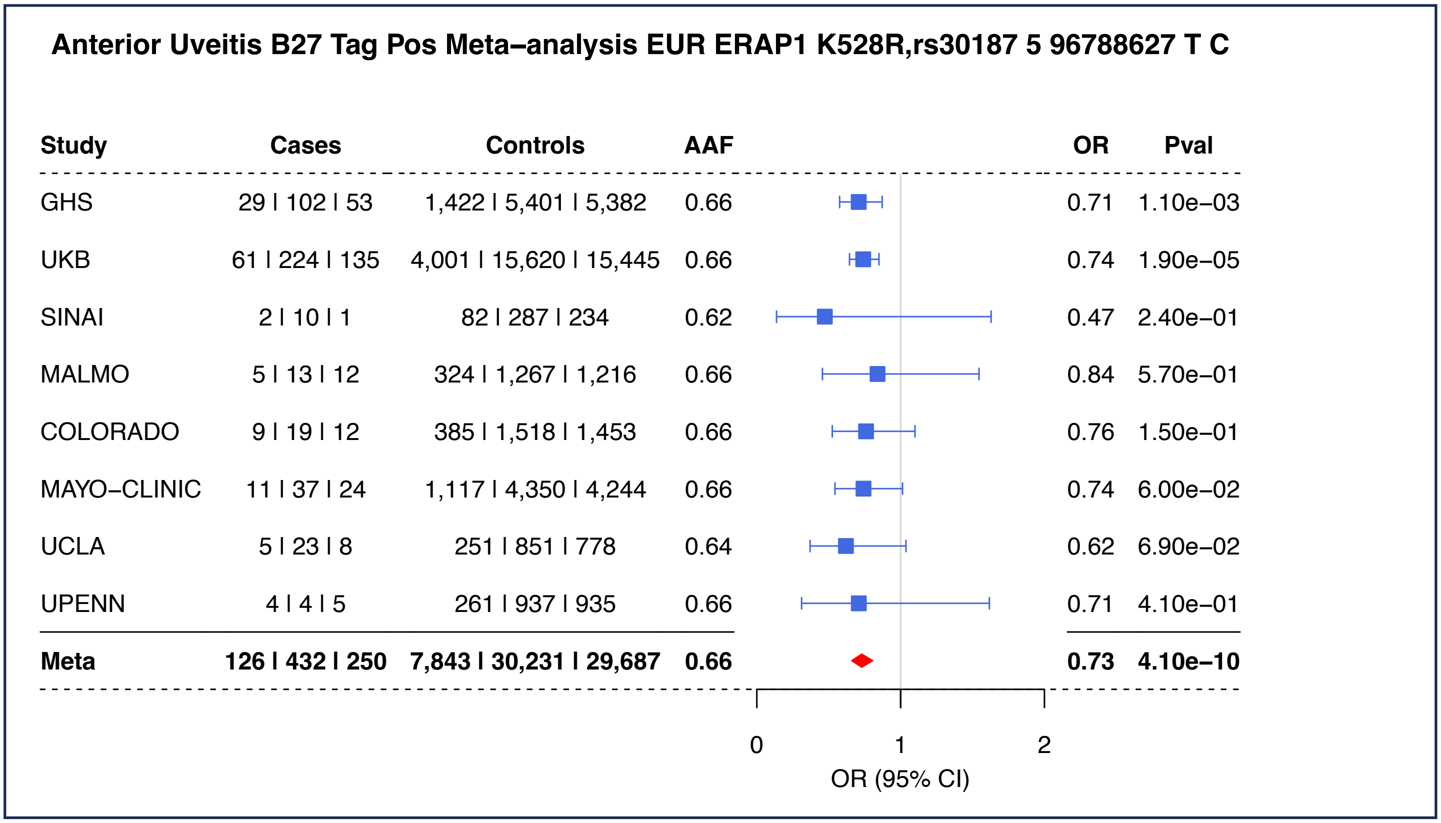

Supplementary Figure S6. Strict B*27-pos analysis removing all samples diagnosed with either AS or psoriasis. A locus zoom plot depicting the -log10(P-value) for all common variants (y-axis) across the ERAP1 region (x-axis) in an analysis considering only B*27 carriers that were not diagnosed with either AS or psoriasis, including 618 AU cases and 67,256 controls. Association models were run with age, age^2, sex and age × sex, and 10 ancestry-informative principal components as covariates. P-values are uncorrected and are from two-sided tests performed using approximate Firth logistic regression.

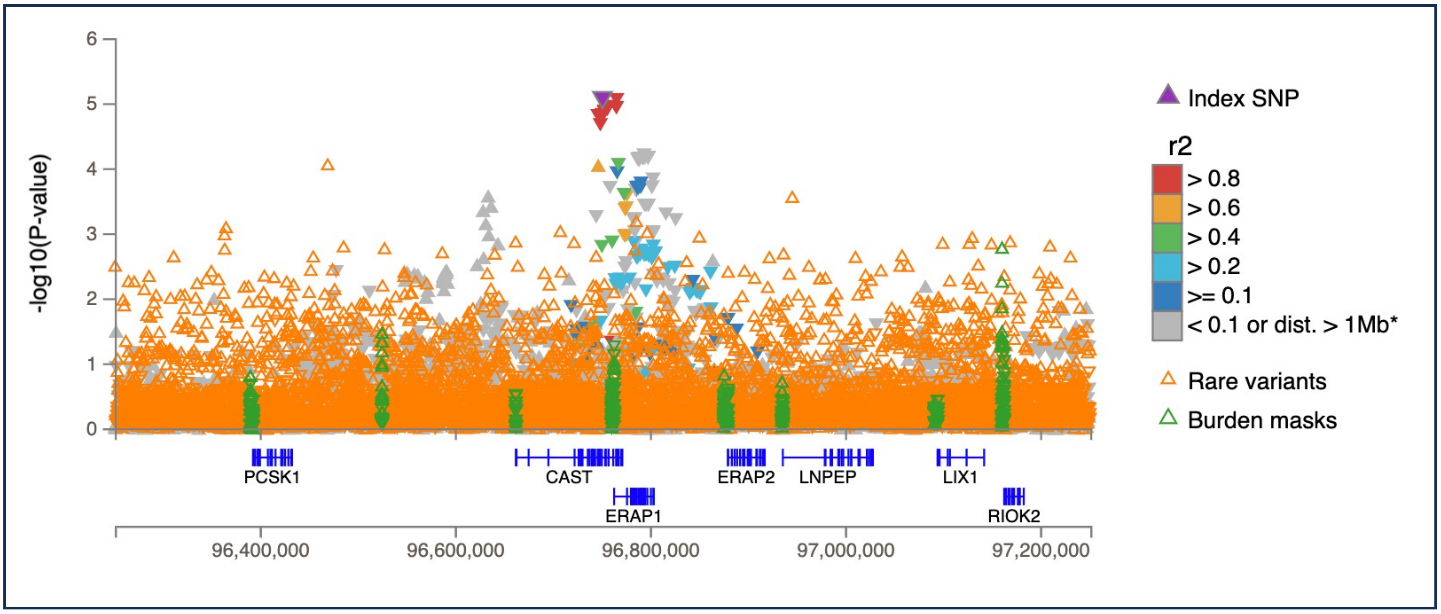

Supplementary Figure S7. Fine-mapping of the *HLA-DPB1* region in a B*27-negative analysis. A locus zoom plot of the *HLA-DPB1* region is presented in top panel. Color represents *r*2 values to the lead variant. The middle and lower panels show posterior inclusion probability (PIP) and conditional PIP (CPIP) values obtained using the Sum of Single Effects (SuSiE) model. A long stretch of non-coding variants that share similar PIPs is observed downstream to *HLA-DPB1*. Association models were run with age, age^2, sex and age × sex, and 10 ancestry-informative principal components as covariates. P-values are uncorrected and are from two-sided tests performed using approximate Firth logistic regression.

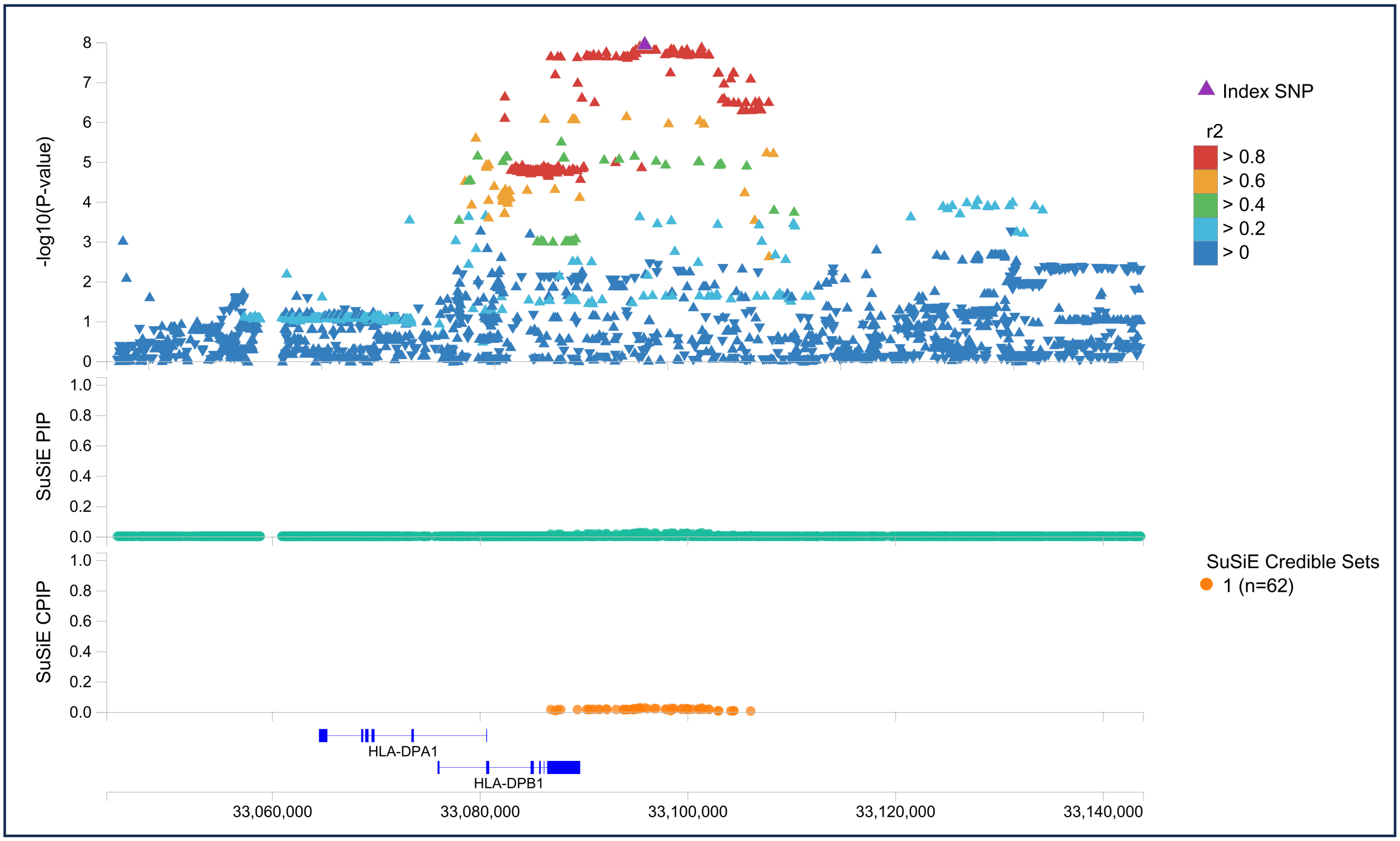

Supplementary Figure S8. Single-Cell Expression of *IPMK* in tissues of the Ciliary body. Violin plots depicting *IPMK* expression distribution across Ciliary body tissues obtained from the Cell atlas of the human ocular anterior segment, obtained and profiled from non-diseased anterior segment tissues from 6 human donors.

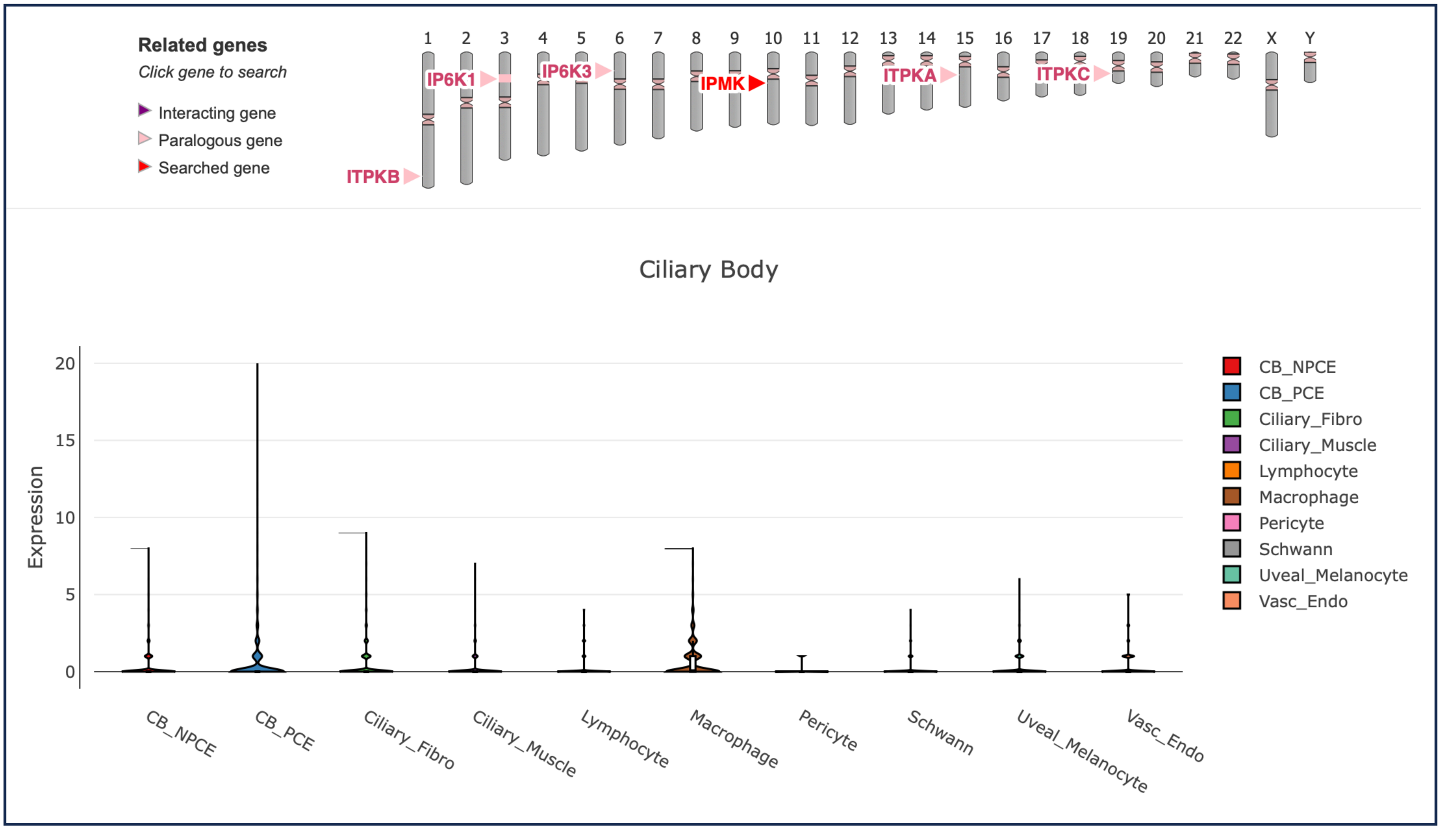

Supplementary Figure S9. Single-Cell Expression of *IPMK* in tissues of the Iris. Violin plots depicting *IPMK* expression distribution across Iris tissues obtained from the Cell atlas of the human ocular anterior segment, obtained and profiled from non-diseased anterior segment tissues from 6 human donors.

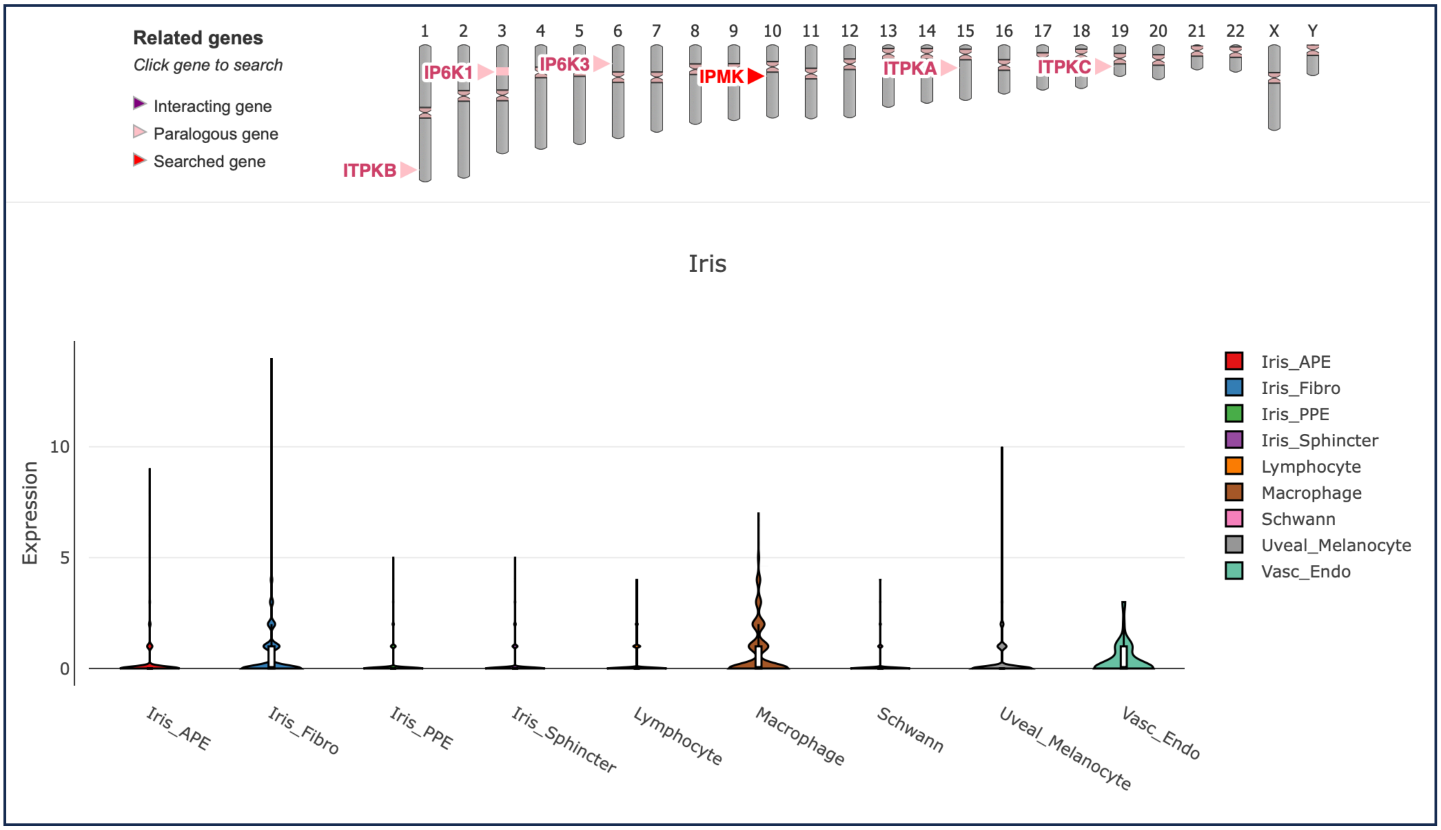

Supplementary Figure S10. Single-Cell Expression of *IDO2* in tissues of the Ciliary body. Violin plots depicting *IDO2* expression distribution across Ciliary body tissues obtained from the Cell atlas of the human ocular anterior segment, obtained and profiled from non-diseased anterior segment tissues from 6 human donors.

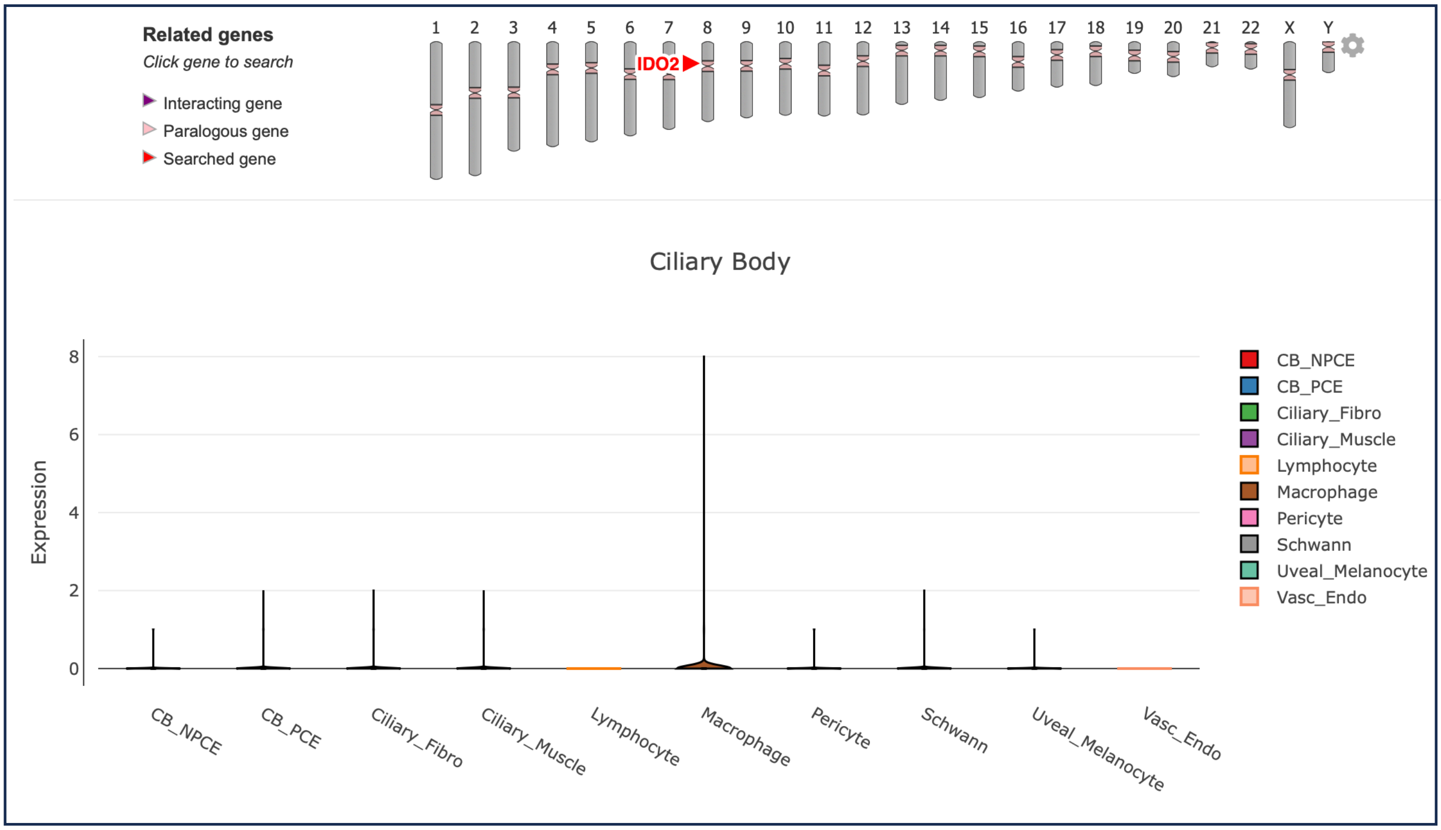

Supplementary Figure S11. Single-Cell Expression of *IDO2* in tissues of the Iris. Violin plots depicting *IDO2* expression distribution across Iris tissues obtained from the Cell atlas of the human ocular anterior segment, obtained and profiled from non-diseased anterior segment tissues from 6 human donors.

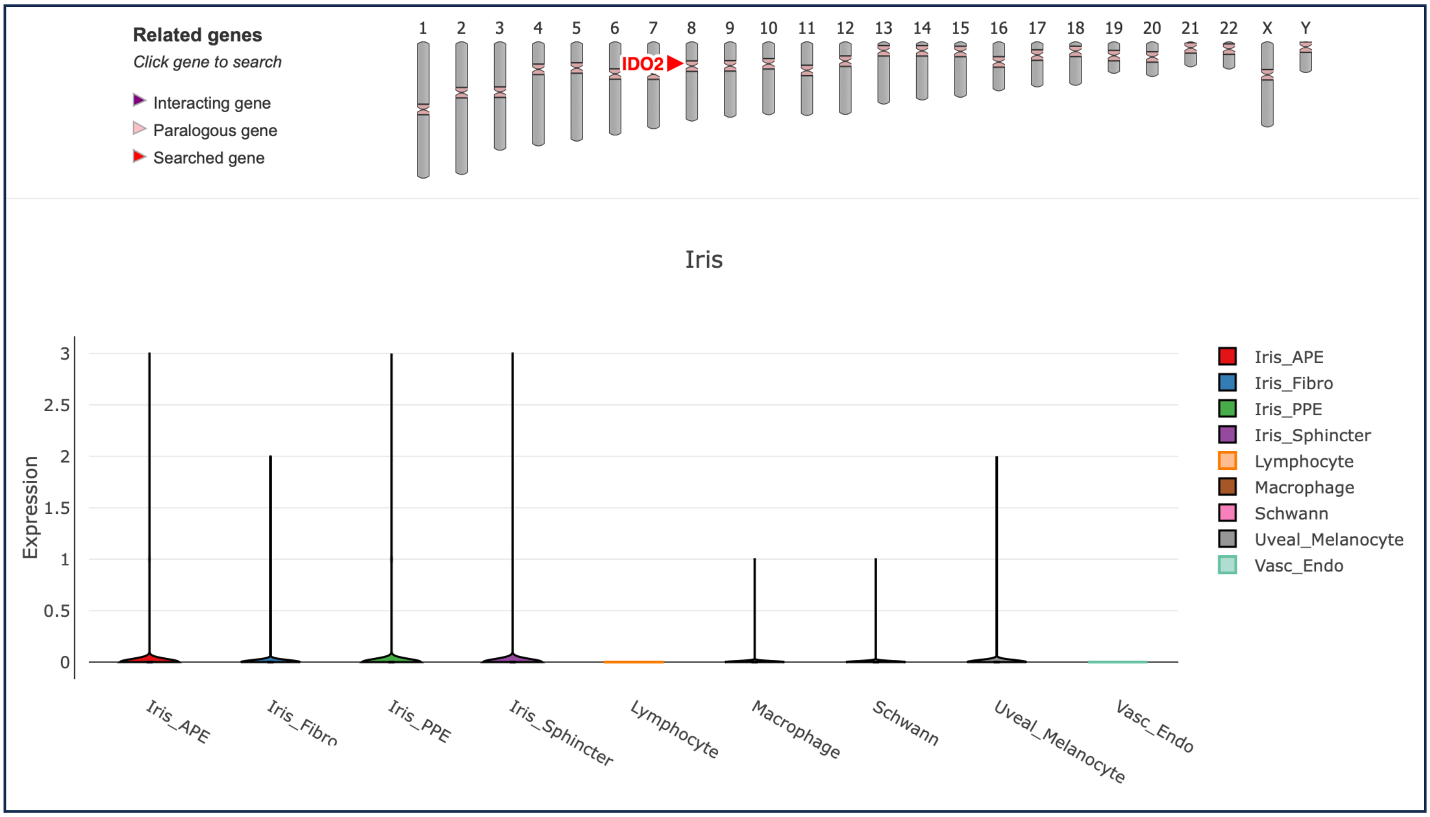

Supplementary Figure S12. Ancestry Specific Forest Plots of *ACHE, STXBP2* and *ADGRF5*. A) A forest plot of EUR, AFR and ALL ancestries is presented for *ACHE* gene, showing that all six carriers are of EUR ancestry. B) A forest plot of EUR, AFR and ALL ancestries is presented for *STXBP2* gene, showing that all nine carriers are of AFR ancestry. C) A forest plot of EUR, AFR and ALL ancestries is presented for *ADGRF5* gene, showing that nominal risk is maintained for both EUR and AFR ancestries, where four out of five carriers are of EUR ancestry, and one additional carrier is of AFR ancestry. Forest plots are sorted by OR (Column 2).

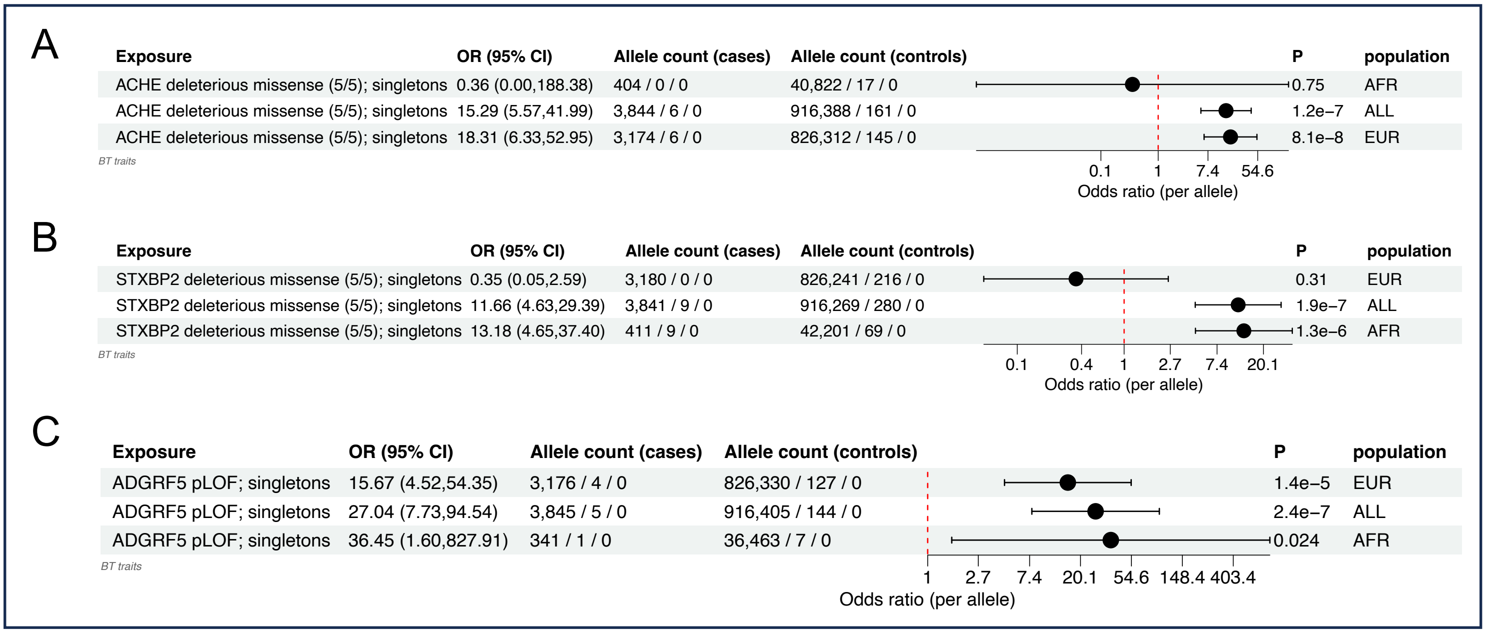

Mayo Clinic Project Generation (PG) Banner Author List and Contribution Statements

PG Leadership Team
Cerhan, James R., M.D.; Couch, Fergus J., Ph.D.

Statistical Genetics and Bioinformatics
Larson, Nicholas B., Ph.D., M.S.; Klee, Eric W., Ph.D.; Fredericksen, Zachary S.; Hart, Steven N., Ph.D.; Lauer, Kimberly P.

Clinical Informatics
Liu, Hongfang, Ph.D.; Wen, Andrew, M.S.

Laboratory Operations
Cicek, Mine, Ph.D.

Registry Principal Investigators
(Alphabetical listing)

1. Alcohol Use Disorder (AUD): Biernacka, Joanna M., Ph.D.
2. Alzheimer's Disease Research Center (ADRC): Vemuri, Prashanthi, Ph.D.; Ramanan, Vijay K., M.D., Ph.D.
3. Bipolar disorder registry: Biernacka, Joanna M., Ph.D.
4. Brain: Jenkins, Robert B., M.D., Ph.D.
5. Breast - Mayo Florida: McLaughlin, Sarah A., M.D.
6. Breast - Mayo Rochester: Olson, Janet E., Ph.D.; Couch, Fergus J., Ph.D.
7. Cardiovascular Disease Specimen Repository: Bielinski, Suzette J., Ph.D., M.Ed.
8. Chronic Kidney Disease: Lieske, John C., M.D.
9. Chronic Pain: Hooten, W. Michael, M.D.
10. Colorectal: Boardman, Lisa A., M.D.
11. COVID-19 Biobank: Kennedy, Richard B., Ph.D.; Cerhan, James R., M.D., Ph.D.; Badley, Andrew D., M.D.
12. Endometrial: Dowdy, Sean C., M.D.; Bakkum-Gamez, Jamie N., M.D.; Glaser, Gretchen E., M.D.
13. Lung: Yang, Ping, M.D., Ph.D.
14. Lymphoma: Cerhan, James R., M.D., Ph.D.
15. Mayo Clinic Biobank: Olson, Janet E., Ph.D.
16. Mayo Clinic Study of Aging (MCSA): Vemuri, Prashanthi, Ph.D.; Ramanan, Vijay K., M.D., Ph.D.
17. Mayo Mammography Health Study: Vachon, Celine M., Ph.D.
18. Multiple Myeloma (MM)/Smouldering MM (SMM): Dispenzieri, Angela, M.D.; Vachon, Celine M., Ph.D.
19. Neuroendocrine pancreas: Antwi, Samuel O., Ph.D.
20. Ovarian: Kaufmann, Scott H.,M.D., Ph.D.; Goode, Ellen L., Ph.D.
21. Pancreas adenocarcinoma: Antwi, Samuel O., Ph.D.
22. Parkinson’s Disease: Ahlskog, J. Eric, M.D., Ph.D.; Bower, James H., M.D.
23. Polycystic kidney disease: Harris, Peter C., Ph.D.
24. Polyps: Boardman, Lisa A., M.D.
25. Prevalence of Asymptomatic Ventricular Dysfunction: Pereira, Naveen L., M.D.
26. PRISM Mammography study: Couch, Fergus J., Ph.D.; Vachon, Celine M., Ph.D.; Liu, Minetta C., M.D.; Olson, Janet E., Ph.D.
27. Prostate: Mine Cicek
28. Prostate Family: Cicek, Mine, Ph.D.
29. Radiation Oncology Registry: Ma, Daniel J., M.D.; Mutter, Robert W., M.D.
30. Renal: Eckel Passow, Jeanette E., Ph.D.
31. Vascular Diseases Biorepository: Kullo, Iftikhar J., M.D.

Management
Danielsen, Andrew J.; Harrington, Jonathan J.; Kushwaha, Jennifer M.

Penn Medicine BioBank Banner Author List and Contribution Statements

PMBB Leadership Team
Daniel J. Rader, M.D., Marylyn D. Ritchie, Ph.D.

Patient Recruitment and Regulatory Oversight
JoEllen Weaver, Nawar Naseer, Ph.D., M.P.H., Afiya Poindexter, Khadijah Hu-Sain, Yi-An Ko, Ph.D.

Lab Operations
JoEllen Weaver, Meghan Livingstone, Fred Vadivieso, Stephanie DerOhannessian, Teo Tran, Julia Stephanowski, Monica Zielinski, Ned Haubein, Joseph Dunn

Clinical Informatics
Anurag Verma, Ph.D., Colleen Morse Kripke, M.S. DPT, MSA, Marjorie Risman, M.S., Renae Judy, B.S.

Genome Informatics
Anurag Verma Ph.D., Shefali S. Verma, Ph.D., Yuki Bradford, M.S., Scott Dudek, M.S., Theodore Drivas, M.D., Ph.D.

UCLA ATLAS banner author list

Chang TS
Ding Y
Freund MK
Johnson R
Schwarz T
Yabu JM
Hazlett C
Chiang JN
Wulf DA
Geschwind DH
Butte MJ
Pasaniuc B
Lajonchere C
Naeim A
Dry S
Wenger N
Elashoff D
Vangala S
Petruse A
Ariannejad M
Magyar C
Johansen L
Werre G
Kroloff M

GHS DiscovEHR Collaboration banner author list

Adam Buchanan	Geisinger Health System, Danville, PA, USA
David J. Carey	Geisinger Health System, Danville, PA, USA
Christa L. Martin	Geisinger Health System, Danville, PA, USA
Michelle Meyer	Geisinger Health System, Danville, PA, USA
Kyle Retterer	Geisinger Health System, Danville, PA, USA
David Rolston	Geisinger Health System, Danville, PA, USA

Colorado Center for Personalized Medicine banner author list

Christina L Aquilante<br>Lisen Axell<br>Kathleen C Barnes<br>Ian M Brooks<br>Tonya M Brunetti<br>Emily Casteel<br>Sameer Chavan<br>Marilyn Coors<br>Kristy Crooks<br>Michelle N Edelmann<br>Matthew Fisher<br>Debashis Ghosh<br>Christopher R Gignoux<br>Casey S Greene<br>Emily Hearst<br>Steve Hess<br>Madelyne Hull<br>Jean Jirikowic<br>Michael G Kahn<br>David Kao<br>Lisa Ku<br>Elizabeth Kudron<br>Yee Ming Lee<br>JosephLesny<br>Meng Lin<br>Jan Lowery<br>Rasika Mathias<br>David A Mayer<br>Ethan Moore<br>JoshuaL Morgenstern<br>J. Tacker Patton<br>Brett Peterson<br>Tzu L Phang<br>Nikita Pozdeyev<br>Nicholas Rafaels<br>Emily R Roberts<br>Alanna N Roff<br>Elise L Shalowitz<br>Jonathan A Shortt<br>Harry Smith<br>Adrian Stewart<br>Anna Tanaka<br>Matthew RG Taylor<br>Emily Todd	Sarah White<br>Stephen J Wicks<br>Laura K Wiley Cole Williams<br>Melissa P Wilson<br>Richard Zane
