# Supplementary material for: A large meta-analysis identifies genes associated with anterior uveitis
Source: Nat Commun. 2023 Nov 11;14:7300. doi: 10.1038/s41467-023-43036-1 (PMC10638276; doi:10.1038/s41467-023-43036-1)
Supplement: Supplementary file 3 — Description of Additional Supplementary Files [file 41467_2023_43036_MOESM3_ESM.pdf]

Description of Additional Supplementary Files

File Name: Supplementary Data 1
Description: Single-variants that are included in the *IPMK* PLoF and damaging missense (5/5) AF<0.1% gene burden mask.

File Name: Supplementary Data 2
Description: Annotated list of variants in AU cases that are included in the top gene burden results.

File Name: Supplementary Data 3
Description: Single-variants that are included in the *IDO2* PLoF AF<0.1% gene burden mask.

File Name: Supplementary Data 4
Description: *ERAP1* haplotype associations with B27-positive AU.

File Name: Supplementary Data 5
Description: The combined risk for AU with *HLA-B27* and *ERAP1*-haplotyes.

File Name: Supplementary Data 6
Description: HLA Class-II allele associations for B27-negative cohort.

File Name: Supplementary Data 7
Description: HLA Class-II allele associations for B27-negative cohort, adjusted for top *HLA-DPB1* SNP.

File Name: Supplementary Data 8
Description: Rare gene burden masks significantly associated with B27-negative AU.

File Name: Supplementary Data 9
Description: Cohort breakdown and *HLA-B27* carrier counts.
