# Supplementary material for: A large meta-analysis identifies genes associated with anterior uveitis
Source: Nat Commun. 2023 Nov 11;14:7300. doi: 10.1038/s41467-023-43036-1 (PMC10638276; doi:10.1038/s41467-023-43036-1)
Supplement: Supplementary file 13 — Reporting Summary [file 41467_2023_43036_MOESM13_ESM.pdf]

Corresponding author(s): Sahar Gelfman

Last updated by author(s): Sep 25, 2023

# Reporting Summary

Nature Portfolio wishes to improve the reproducibility of the work that we publish. This form provides structure for consistency and transparency in reporting. For further information on Nature Portfolio policies, see our Editorial Policies and the Editorial Policy Checklist.

## Statistics

For all statistical analyses, confirm that the following items are present in the figure legend, table legend, main text, or Methods section.

| n/a | Confirmed | |
|---|---|---|
| ☐ | ☒ | The exact sample size (*n*) for each experimental group/condition, given as a discrete number and unit of measurement |
| ☐ | ☒ | A statement on whether measurements were taken from distinct samples or whether the same sample was measured repeatedly |
| ☐ | ☒ | The statistical test(s) used AND whether they are one- or two-sided<br>*Only common tests should be described solely by name; describe more complex techniques in the Methods section.* |
| ☐ | ☒ | A description of all covariates tested |
| ☐ | ☒ | A description of any assumptions or corrections, such as tests of normality and adjustment for multiple comparisons |
| ☐ | ☒ | A full description of the statistical parameters including central tendency (e.g. means) or other basic estimates (e.g. regression coefficient) AND variation (e.g. standard deviation) or associated estimates of uncertainty (e.g. confidence intervals) |
| ☐ | ☒ | For null hypothesis testing, the test statistic (e.g. *F*, *t*, *r*) with confidence intervals, effect sizes, degrees of freedom and *P* value noted<br>*Give P values as exact values whenever suitable.* |
| ☒ | ☐ | For Bayesian analysis, information on the choice of priors and Markov chain Monte Carlo settings |
| ☐ | ☒ | For hierarchical and complex designs, identification of the appropriate level for tests and full reporting of outcomes |
| ☒ | ☐ | Estimates of effect sizes (e.g. Cohen's *d*, Pearson's *r*), indicating how they were calculated |

*Our web collection on statistics for biologists contains articles on many of the points above.*

## Software and code

Policy information about availability of computer code

| | |
|---|---|
| Data collection | No software was used for data collection. |
| Data analysis | The REGENIE software for whole genome regression, which was used to perform all genetic association analysis, is available at https://github.com/rgcgithub/regenie. GCTA v1.91.7 was used for approximate conditional analysis. SHAPEIT4.2.0 was used for phasing of SNP array data. Imputation was completed with IMPUTE5. We use Plink1.9/2.0 for genotypic analysis. FINEMAP 1.4.1 and SuSiE 0.12.27 were used for fine-mapping, and genetic correlations were calculated using LDSC version 1.0.1 with annotation input version 2.2. R Statistical Computing 4.1 was used including packages with visualization tools, statistical and data processing libraries (e.g. base R 4.1, dplyr 1.1.2, ggplot2 3.3.6, data.table 1.14.2). |

For manuscripts utilizing custom algorithms or software that are central to the research but not yet described in published literature, software must be made available to editors and reviewers. We strongly encourage code deposition in a community repository (e.g. GitHub). See the Nature Portfolio guidelines for submitting code & software for further information.

## Data

Policy information about availability of data

All manuscripts must include a data availability statement. This statement should provide the following information, where applicable:

- Accession codes, unique identifiers, or web links for publicly available datasets
- A description of any restrictions on data availability
- For clinical datasets or third party data, please ensure that the statement adheres to our policy

The primary analysis summary data generated in this study have been deposited in the GWAS Catalog database under accession code [GCST ID: GCST90295958, GCST90295959]. Individual-level sequence data have been deposited with UK Biobank and will be freely available to approved researchers, as done with other genetic datasets to date. Individual-level phenotype data are already available to approved researchers for the surveys and health-record datasets from which all our traits are derived. Instructions for access to UK Biobank data is available at https://www.ukbiobank.ac.uk/enable-your-research. Summary statistics from UK Biobank trait are available in the GWAS Catalog (accession IDs are listed in the tables description sheet available in the supplementary data tables excel file). Exome sequencing and genotyping data used for meta-analysis from additional cohorts such as the Geisinger Health System MyCode, the Malmö Diet and Cancer Study, the University of Pennsylvania Penn Medicine BioBank, the Mount Sinai BioMe BioBank and the Colorado Center for Personalized Medicine Biobank, can be made available to qualified, academic, non-commercial researchers upon request via a Data Transfer Agreement with the respective research institute. Aggregate data from the UCLA ATLAS Community Health Initiative can be made available to qualified, academic, non-commercial researchers on a collaborative basis upon request. As described in Backman et al.9, the HapMap3 reference panel was downloaded from https://ftp.ncbi.nlm.nih.gov/hapmap/, GnomAD v3.1 VCFs were obtained from https://gnomad.broadinstitute.org/downloads, and VCFs for TOPMED Freeze 8 were obtained from dbGaP as described in https://topmed.nhlbi.nih.gov/topmed-whole-genome-sequencing-methods-freeze-8.

## Research involving human participants, their data, or biological material

Policy information about studies with human participants or human data. See also policy information about sex, gender (identity/presentation), and sexual orientation and race, ethnicity and racism.

| | |
|---|---|
| Reporting on sex and gender | Neither sex nor gender were considered in the study design. Analyses were not stratified by sex, although genetically determined sex was used as a covariate in the GWAS. |
| Reporting on race, ethnicity, or other socially relevant groupings | We performed African (AFR) and European (EUR) ancestry specific analyses using ancestry labels derived from the genetic data. Ancestries were defined by genetic principal components derived from the hapmap population reference. Genetic principal components derived from the data were further used as covariates in all analyses. |
| Population characteristics | The UK Biobank is a prospective cohort study previously described in detail by Bycroft et al, Nature 2018 (https://www.nature.com/articles/s41586-018-0579-z). Briefly, 94.7% of sequenced participants are of European ancestry, 54.2% are female, the average age at assessment is 58, and the mean BMI is 26. 45% of participants report a history of smoking, and each participant reports 8 inpatient ICD10 3D codes, on average. See supplementary table 1 for additional details. |
| Recruitment | Please see Bycroft et al, Nature 2018. |
| Ethics oversight | Ethical approval for the UK Biobank was previously obtained from the North West Centre for Research Ethics Committee (11/ NW/0382). The work described herein was approved by UK Biobank under application number 26041. Approval for Geisinger Health System MyCode analyses was provided by the Geisinger Health System Institutional Review Board under project number 2006-0258. Informed consent was obtained for all study participants. Appropriate consent for the University of Pennsylvania Penn Medicine BioBank was obtained from each participant regarding storage of biological specimens, genetic sequencing and genotyping, and access to all available EHR data. This study was approved by the Institutional Review Board of the University of Pennsylvania and complied with the principles set out in the Declaration of Helsinki. All subjects participating in the MAYO-RGC Project Generation provided informed consent for use of specimens and data in genetic and health research and ethical approval for Project Generation was provided by the Mayo Clinic IRB (#09-007763). Ethical approval and consent for the Colorado Center for Personalized Medicine Biobank was reviewed and approved by the Colorado Multiple Institutional Review Board (#15-0461). All research performed in the UCLA ATLAS Community Health Initiative study conformed with the principles of the Helsinki Declaration. All individuals provided written informed consent to the original recruitment of the UCLA ATLAS Community Health Initiative. Patient Recruitment, Sample Collection for Precision Health Activities at UCLA is an approved study by the UCLA Institutional Review Board (UCLA IRB). IRB#17-001013. All research performed in this study uses de-identified data (without any Protected Health Information data) with no possibility of re-identifying any of the participants. The Mount Sinai BioMe BioBank study protocols were approved by the institutional review board of the Icahn School of Medicine at Mount Sinai. Written informed consent was obtained for all study participants. All participants in the Malmö Diet and Cancer Study were provided written informed consent and the study was approved by the Lund University Ethics Committee (MDC LU 51-90) and for the cadmium sub-study (2009/633). |

Note that full information on the approval of the study protocol must also be provided in the manuscript.

# Field-specific reporting

Please select the one below that is the best fit for your research. If you are not sure, read the appropriate sections before making your selection.

☒ Life sciences ☐ Behavioural & social sciences ☐ Ecological, evolutionary & environmental sciences

# Life sciences study design

All studies must disclose on these points even when the disclosure is negative.

| | |
|---|---|
| Sample size | Sample size was not predetermined. Association analyses were restricted to the intersection of samples with both exome sequence and array genotypes available after QC. See methods section "Exome sequencing" for details on QC performed. All samples that pass genotype QC and with non-missing phenotype data were included in association analyses. Sample sizes represent all available samples from all eight cohort, which together represent a significant increase in sample size relatively to prior publications in the literature. |
| Data exclusions | Phenotype selection and QC was performed as described in methods section "Health- and behavior-related phenotypes." Variant level QC was performed as described in methods section "Exome sequencing." The minor allele count threshold was pre-determined based on extensive simulations performed with REGENIE. See https://www.nature.com/articles/s41588-021-00870-7 for additional details. |
| Replication | We did not have a separate replication cohort internally. We pooled genetic data from all our internal cohorts (UKB, GHS, UPenn-PMBB, Sinai, MALMO, Colorado, UCLA, MAYO) to perform a meta-analysis. We identified several significant loci and rare gene burdens including ERAP1, HLA-B, IPMK and IDO2, for which we looked for consistency in the effect size directions and evidence for statistical significance in the individual cohorts. The meta-analysis and individual cohort results of the common loci are reported in Figure 2 and Figure 3.<br>For all significant genes/loci, we observed a consistent direction of effect in all cohorts with at least one case carrier (at least three cohorts for rare burdens) and both a consistent direction of effect and statistical significance in at least two cohorts. |
| Randomization | Randomization was not required for the analyses completed in this study. To control for confounding, we performed association analysis with the following covariates included in the regression model: age, age-squared, sex, age-x-sex, 10 ancestry-informative principal components, six exome sequence batch indicator variables, and 20 principal components derived from exome variants with a MAF between 2.6x10-5 and 1%. |
| Blinding | Blinding is not required in this study as the phenotyping, genotyping and statistical analyses are completely independent processes and each happened without any prior knowledge of the others. |

# Reporting for specific materials, systems and methods

We require information from authors about some types of materials, experimental systems and methods used in many studies. Here, indicate whether each material, system or method listed is relevant to your study. If you are not sure if a list item applies to your research, read the appropriate section before selecting a response.

## Materials & experimental systems

| n/a | Involved in the study | |
|---|---|---|
| ☒ | ☐ | Antibodies |
| ☒ | ☐ | Eukaryotic cell lines |
| ☒ | ☐ | Palaeontology and archaeology |
| ☒ | ☐ | Animals and other organisms |
| ☒ | ☐ | Clinical data |
| ☒ | ☐ | Dual use research of concern |
| ☒ | ☐ | Plants |

## Methods

| n/a | Involved in the study | |
|---|---|---|
| ☒ | ☐ | ChIP-seq |
| ☒ | ☐ | Flow cytometry |
| ☒ | ☐ | MRI-based neuroimaging |
